# Gas Sensors Based on Polymer Field-Effect Transistors

**DOI:** 10.3390/s17010213

**Published:** 2017-01-22

**Authors:** Aifeng Lv, Yong Pan, Lifeng Chi

**Affiliations:** 1 Physikalisches Institut and Center for Nanotechnology (CeNTech), Universität Münster, Wilhelm-Klemm-Str. 10, Münster 48149, Germany; lva@uni-muenster.de (A.L.); pany@uni-muenster.de (Y.P.); 2 Jiangsu Key Laboratory for Carbon-Based Functional Materials & Devices, Functional Nano and Soft Materials Laboratory (FUNSOM), Soochow University, Renai Rd. 199, Suzhou 215123, China

**Keywords:** OFETs, sensor, gas analyte, polymer, sensing mechanism

## Abstract

This review focuses on polymer field-effect transistor (PFET) based gas sensor with polymer as the sensing layer, which interacts with gas analyte and thus induces the change of source-drain current (Δ*I*_SD_). Dependent on the sensing layer which can be semiconducting polymer, dielectric layer or conducting polymer gate, the PFET sensors can be subdivided into three types. For each type of sensor, we present the molecular structure of sensing polymer, the gas analyte and the sensing performance. Most importantly, we summarize various analyte–polymer interactions, which help to understand the sensing mechanism in the PFET sensors and can provide possible approaches for the sensor fabrication in the future.

## 1. Introduction

Organic field-effect transistor (OFET) based gas sensors have attracted great interest due to the high selectivity, repeatable response and low-cost production [1,2,3,4,5,6]. The organic materials, which interact with gas analytes, can fine-tune their chemical and physical properties via precise organic synthesis. Additionally, covalent integration of recognition groups onto the sensing molecules can provide specific interaction with designated analytes, thus significantly increasing the selectivity and response. The thin films of organic materials can be deposited by printing technology at low temperatures on flexible substrate. As a result, miniaturization and high integration of these sensors at low cost are achievable. Moreover, the device configuration of OFETs and the instrument for the response measurement are relatively simple. These advantages endow the OFET sensors with great potential application in electronic nose, which can reproduce the human sensing with sensor arrays and pattern recognition systems. Such electronic nose is similar to human olfaction, which has applications of identification, comparison, quantification, data storage and retrieval.

**Scheme 1:**
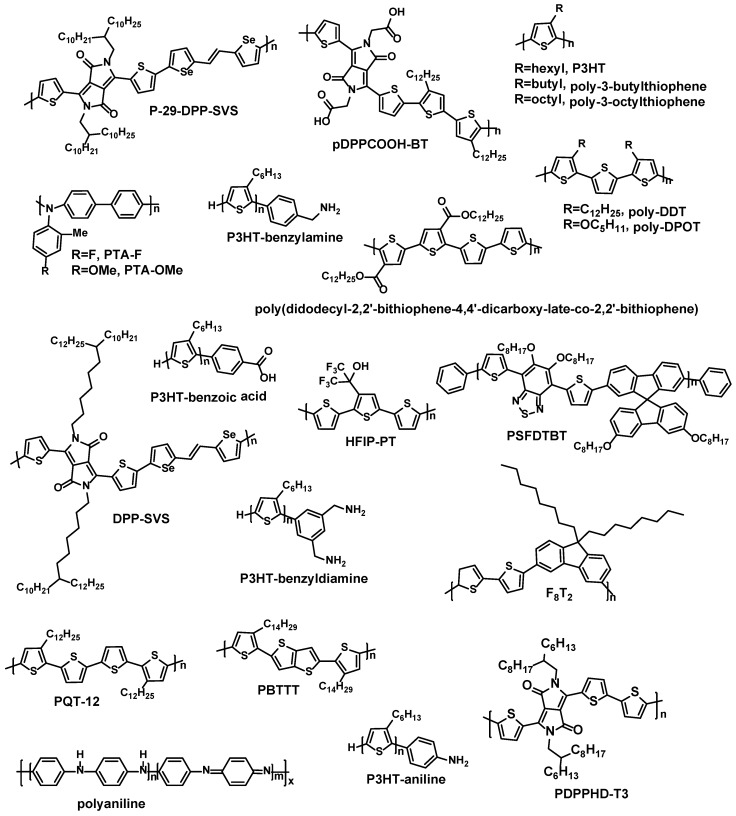
Reported molecular structures of semiconducting polymer used in the OFET sensors.

There are mainly two types of organic electrical sensors: chemiresistor and OFET. Chemiresistor sensors exhibit change of resistance toward gas analytes that have not yet reached satisfactory performance. OFET sensors become more competitive due to the ability of delivering multi-parameter response, for example, charge carrier mobility (*μ*), threshold voltage (*V*_T_), on/off-current (*I*_ON_/*I*_OFF_) of the devices, and the bulk conductivity (*σ*) of the organic semiconducting film [7,8]. Such devices can display fingerprint response for the detection of a wide range of analytes at low concentration. In an OFET sensor, charge carrier mobility is a very important factor as it relates with the signal transfer speed [9]. Therefore, OFET sensors based on the small molecular semiconductors have attracted great scientific interest due to the crystalline packing in the thin film and the resulting high charge carrier mobility. However, small molecular semiconductors are normally fabricated in a high-vacuum set-up by which the fabrication process is expensive and time-consuming. On the opposite side, semiconducting polymers have cost effectiveness, easy processing and compatibility with plastic substrate, and therefore have great advantageous applications in the electronic nose. However, OFET sensors have not been conducted on many existing polymer semiconductors (Scheme 1, Table 1), and previous literature has mainly focused on the research of charge carrier transport [10].

**Figure 1:**
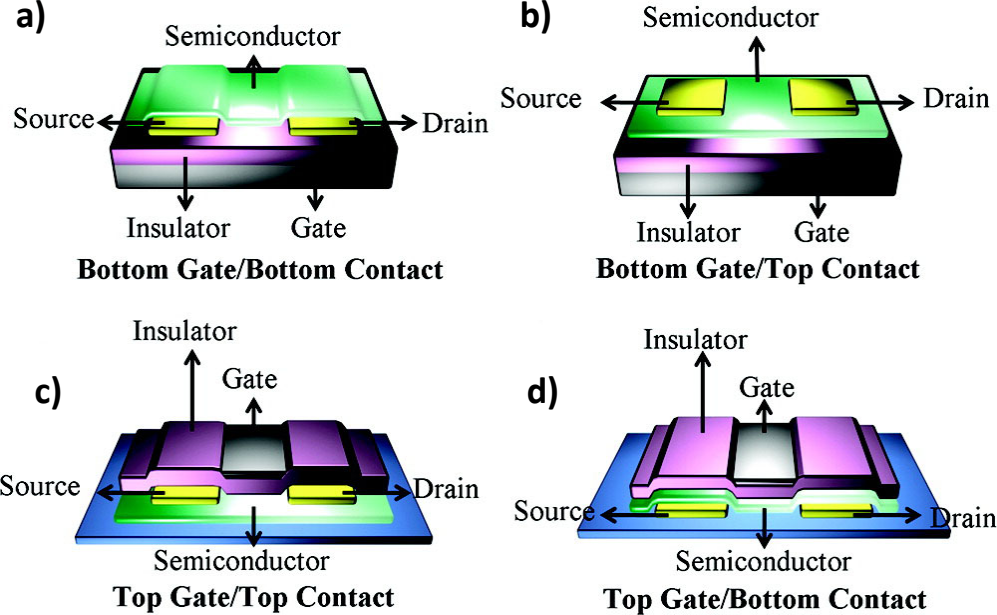
Four types of OFET device structure: (**a**) bottom gate/bottom contact; (**b**) bottom gate/top contact; (**c**) top gate/top contact; and (**d**) top gate/bottom contact. Reprinted with permission from Ref. [40]. Copyright (2012) American Chemical Society.

Here, we only review the sensors operated as transistors with source, drain and gate electrodes and with organic polymer as the semiconducting layer. There are usually four transistor configurations for sensor applications, as shown in Figure 1 [40]. Bottom gate/bottom contact (BGBC) and bottom gate/top contact (BGTC) are mostly applied in the OFET sensors while top gate/top contact (TGTC) and top gate/bottom contact (TGBC) are usually used in the metal-oxide-semiconductor field-effect transistor (MOSFET) sensors. It is important to distinguish the layer (sensing layer) that interacts with gas analytes among polymer semiconducting layer, dielectric layer and conductive polymer gate. According to the sensing layer, the sensors can be further subdivided into three types of sensors, for which this review focuses on the sensing molecules, vapor analytes and sensing performance [41,42]. We have also tried to understand the interactions between sensing polymers and electrically neutral gas analytes, which have aroused the change of source-drain current (*I*_SD_) in the OFET gas sensors.

## 2. Polymer Field-Effect Transistor (PFET) Sensors with Semiconducting Polymers as the Sensing Layer

The transduction principle of PFET sensors upon exposure to gaseous analytes is ascribed to the change of current at the semiconducting polymer/dielectric interface (*I*_SD_). The current flowing in the conducting channel can either increase or decrease, which depends on gas–polymer interaction. To date, PFET sensors have been able to detect a wide range of vapors including NH_3_, amines, NO_2_, H_2_S, alcohols, etc. Each analyte shows unique response when exposed to PFET devices.

### 2.1. NH_3_ and Amine Gas Sensors

Ammonia (NH_3_) in air can accelerate the formation of hazy weather, which potentially causes a series of chronic diseases such as asthma, and severe respiratory and lung diseases. Therefore, ammonia detection has attracted great attention and the detection limit has enormously decreased from thousands of ppm to tens of ppb level since first being reported [11].

Ammonia usually decreases the flowing current (*I*_SD_) in the device channel. A semiconducting polymer P-29-DPP-SVS has a hole charge carrier mobility around 3.0 cm^2^·V^−1^·s^−1^ which is high enough to achieve pleasant sensitivity [20]. The OFET devices were then fabricated and succeeded in detecting ammonia in the range of 29 to 1000 ppm at a source-drain voltage (*V*_DS_) of −5 V and gate voltage (*V*_GS_) of −80 V. The lowest detection concentration was 29 ppm which was already much lower than electrical chemiresistor sensors [43]. However, the sensing response toward 29 ppm ammonia is nearly ignorable, which is far from real application. One efficient way to improve the sensitivity is chemical structure adjustment of semiconducting polymer [21]. For example, transformation of ester group (−COOR) into carboxyl group (–COOH) group in the side chain of pDPPCOOH-BT by thermal treatment endows the OFET sensor with good selectivity toward ammonia and amines. The transformation process also accompanies the formation of nanopores in the thin film. The –COOH group and the porous structure make the detection limit go down to 10 ppb at *V*_DS_ = −60 V and *V*_GS_ = −80 V, which is low enough for regular detection.

**Figure 2:**
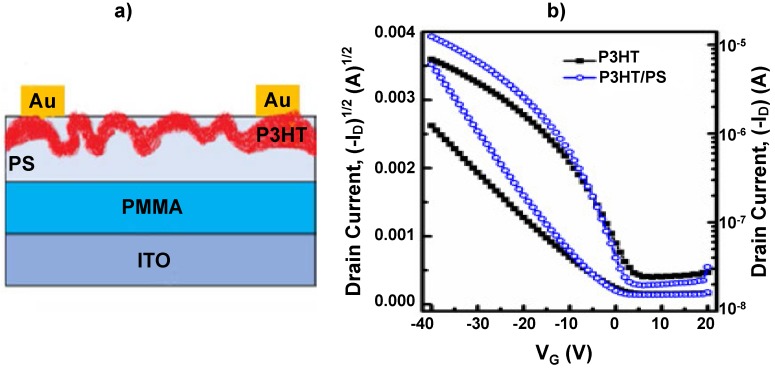
(**a**) Schematic structure of OFET sensor with poly(3-hexylthiophene)/polystyrene (P3HT/PS) blended film; and (**b**) transfer curve of P3HT/PS film with the best mixing ratio of 1:4 in weight. Reprinted with permission from Ref. [22]. Copyright (2016) Elsevier.

Mixing P3HT semiconductor and polystyrene (PS) dielectric created more interfaces to interact with ammonia (Figure 2), which improved analyte–polymer interaction and lowered the detection limit down to 5 ppm at *V*_DS_ = *V*_GS_ = −40 V [22]. The formed P3HT/PS interfaces also made the sensor have superior stability with very little decrease of sensing response even stored in the atmospheric environment for 40 days. Modification on device structure can improve the sensor sensitivity. An interesting work is to replace the gate electrode and dielectric with the introduction of static charges on the back surface of the electret polyethylene terephthalate (PET) substrate by corona charging. This device fabrication process is herein simplified and potentially adaptable to roll-to-roll processing. With *V*_DS_ = −90 V and static gate induced by corona charging, poly(3,3’-didodecylquaterthiophene) (PQT-12) sensor based on this device structure gave a reasonable detection limit of 100 ppb [23]. Vertical transistor, whose current flows through the bulk region in the vertical channel, creates improved interaction between gas analyte and conducting channel. In comparison with P3HT sensor based on traditional transistor (horizontal channel), NH_3_ detection with this device structure obtained a much higher sensitivity with low detection limit of 30 ppb and a low operating voltage around 1 V [12]. Low-voltage operation is required for sensor application in wireless transducer system and portable devices [24].

Amine detection has potential application in food inspection, for instance, putrescine (butane-1,4-diamine) and cadaverine (pentane-1,5-diamine) are salient markers of meat decomposition but discrimination among different amines is hard to realize [44,45]. In contrast to much attention put on ammonia detection, only a few studies are related to amine detection. Besides ammonia, the –COOH group attached on the polymer pDPPCOOH-BT mentioned above also showed good sensitivity toward three amines of 1,4-diaminobutane, Et3N and piperidine, but no discrimination among them [21]. Grazing incidence X-ray diffraction (GIXD) proves that longer side chains result in larger plane spacing in the polythiophene thin films [27]. It is found that longer chains have no dependence on film thickness and usually display stronger response since an analyte can penetrate easily into the polythiophene film. As the side chains shorten, dependence on film thickness increases, especially when the length of the side chain becomes comparable to the size of analyte, usually with 4–5 carbons in the polythiophene chain. By varying side chains of the polythiophene molecule as well as the thickness of the polythiophene films, amines with different molecular size can be differentiated. Sensor array employed permutations between two different side chain lengths (poly-3-butylthiophene and poly-3-octylthiophene) and two different film thicknesses (70 nm and 140 nm) discriminated hexylamine and trimethylamine, which had different molecular size.

**Scheme 2:**
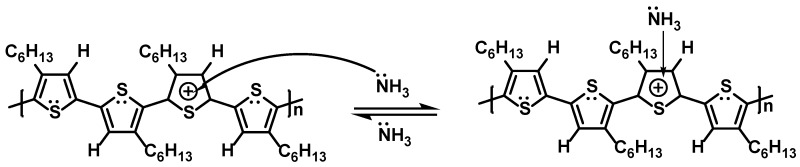
Formation of linkage type structure between NH_3_ and P3HT.

A good understanding of the sensing mechanism is not only important, but may also enable guidelines for modifying and improving sensor performance. Generally, the interaction between gas analyte and sensing layer changes the charge distribution at the semiconductor/dielectric interface. The interaction is always different between various gas analytes and sensing layers and there has been no instruction to predict the analyte–polymer interaction until now. For the tested semiconducting polymers, such as P3HT and its derivatives, PQT-12, pDPPCOOH-BT and so on, ammonia always reduces the current flowing in the conducting channel. The current reduction (Δ*I*_SD_) is generally attributed to lone pair of electrons of ammonia which influences the charge transport in two aspects: (1) form linkage type structure with organic molecule as shown in Scheme 2 when NH_3_ diffuses in the thin film, which causes current decrease in the conducting channel [13]; and (2) trap/dedope at the polymer semiconductor/dielectric interface which negatively shifts the threshold voltage (Δ*V*_T_). NH_3_ dedopes the P3HT film to compensate the ambient oxidant in P3HT film, which has been confirmed in the purification process of synthesized P3HT [13]. Trapping effect of NH_3_ is verified through gas exposure toward P3HT/PS mixture in the film. The mixed film has much P3HT/PS interface and blocks NH_3_ diffusion into the P3HT/dielectric (PMMA) interface, which results in nearly no *V*_T_ shift [22].

Designed functional groups in a polymer molecule play an important role when interacting with gas analyte. The sensing ability of pDPPCOOH-BT OFET sensor is attributed to the −COOH group. Upon exposure to NH_3_, FT-IR signal at 1729 cm^−1^, absorption peak of the −COOH group in pDPPCOOH-BT, was shifted to 1650 cm^−1^, indicating the formation of ammonium carboxylate which might act as additional traps for charge carriers [21].

**Figure 3:**
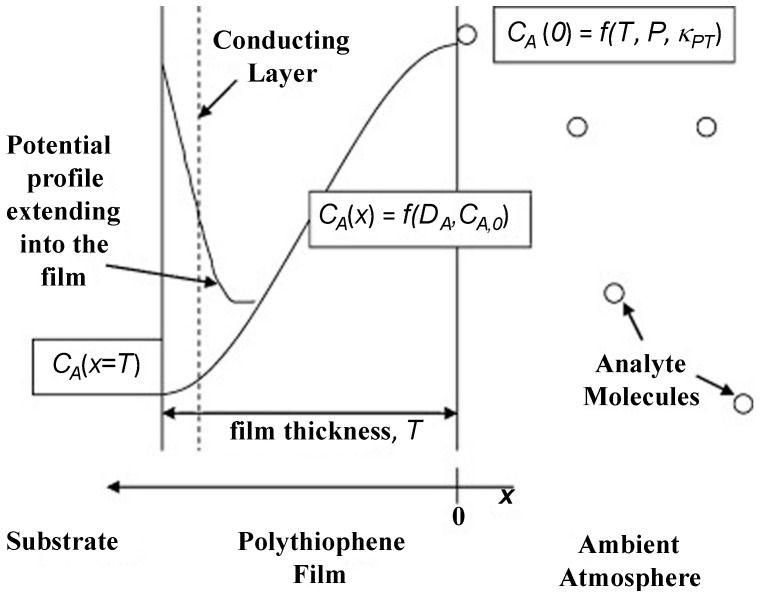
Conceptual framework for physical discrimination. Partial analyte molecules in the ambient atmosphere will adsorb onto the surface of the polythiophene film at *x* = 0 and diffuse into the film toward the substrate, eventually interacting with the semiconductor layer at the dielectric–semiconductor interface. Reprinted with permission from Ref. [27]. Copyright (2010) Elsevier.

There are only a few reports illustrating the interaction between amines and semiconducting polymers. In one study, it is proven that the sensitivity correlates with the molecular volume of amines, which means that larger amines having poor diffusion into the polymer/dielectric interface [27]. They present a framework to describe the analyte concentration from film surface to semiconducting polymer/dielectric interface (Figure 3). In this framework, all factors affecting the analyte concentration are considered. Analyte concentration at the film surface (*C*_A_(0)) is a function of the partition coefficient (*κ*_PT_) of the sensor film, ambient temperature (*K*) and analyte pressure (*P*). Then analytes diffuse into the film until they reach the conducting channel at the semiconducting polymer/dielectric interface. It is the concentration of analyte at this interface, *C*_A_(*x* = T), that gives rise to the electrical response of the sensor. This concentration is dependent on the film thickness (*T*) and the diffusion coefficient (*D*_A_) of the analyte. The diffusion coefficient *D*_A_ encapsulates the effect of film structure, which is a function of analyte size relative to crystalline plane spacing in the film. In another report, X-ray reflectivity (XRR) monitored the poly-3-butylthiophene film swelling under butylamine exposure [46]. This indicates that the film can adsorb analytes and leads to thickness increase [14]. Though the thickness increase is lower than the electrical response, the physical swelling should be included in the sensing mechanisms just like trapping, dedoping and so on.

### 2.2. NO_2_ Gas Sensor

**Figure 4:**
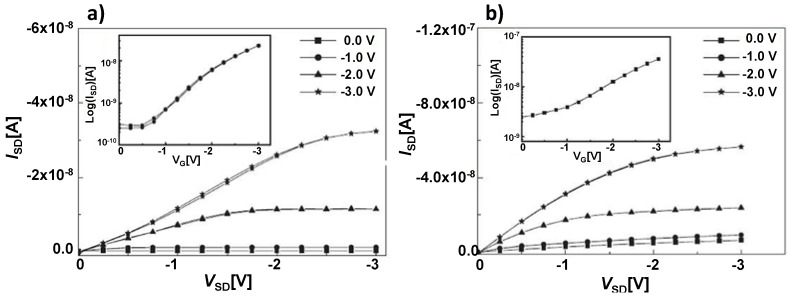
Output curves of PTA-OMe OFETs: (**a**) before NO_2_ exposure; and (**b**) after NO_2_ exposure. The insets show saturated transfer curves of the corresponding OFETs in semilog plots. Reprinted with permission from Ref. [28]. Copyright (2007) Wiley-VCH.

**Scheme 3:**
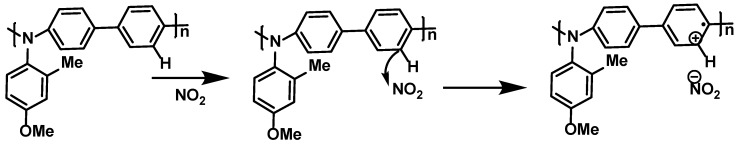
Proposed interaction between NO_2_ and polymer semiconductor PTA-OMe.

Many studies report on NO_2_ gas sensors. The achieved sensitivity is impressive with the lowest detection limit down to 10 ppb [28]. Contrary to ammonia sensor, the NO_2_ gas sensor usually increases the flowing current in the device channel [16,28]. Figure 4 displays the output and transfer characteristics of PTA-OMe OFETs without gas exposure (Figure 4a) and with gas exposure of 10 ppm NO_2_ at *V*_SD_ = *V*_GS_ = −3 V (Figure 4b). There is a clearly nonlinearity of the output characteristic at low source-drain voltage (*V*_SD_) in the pristine device. After exposure to 10 ppm NO_2_, the output characteristics turned linear at low *V*_SD_ and unsaturated at high *V*_SD_, indicating the existence of doping at the contact as well as in the channel. The transfer curve shows the threshold voltage shifted toward positive direction under NO_2_ exposure. The supposed interaction between NO_2_ and PTA-OMe is shown in Scheme 3 [16,47]. In this experiment, semiconducting PTA-OMe was amorphous and had low carrier mobility of around 10^−5^ cm^2^·V^−1^·s^−1^ due to absence of crystalline grain boundary. It is not *μ* but *V*_T_ is more sensitive to NO_2_ gas, which tells the advantage of multi-parameter OFET sensor.

With the same molecular backbone but different substituents in the side chain, PTA-OMe showed higher sensitivity than PTA-F while PTA-F had much shorter recovery time than PTA-OMe. If more attention were paid to the substituents, sensors with high sensitivity as well as fast response are realizable.

### 2.3. Alcohol Sensor

Many alcohol sensors based on pure polymer OFETs have been reported but the sensing response is usually low with detection limit of around thousands of ppm. The sensitivity can be greatly increased by mixing the semiconducting polymer with functionalized graphene oxide (GO) [29,30]. An OFET sensor with mixture of polymer F8T2 and oleylamine-modified GO (OA-GO) as the sensing layer was investigated [30]. In contrast to the pure F8T2, the mixture showed sensitivity 10 times higher in %Δ*V*_T_ and six times higher in %Δ*μ* with respect to ethanol under the same applied voltage of *V*_DS_ = *V*_GS_ = −80 V. The functionalized GO in the composite film helped to adsorb the gas analyte for one side and increased the surface-to-volume ratio for the other side. Moreover, the content of GO in the mixed film should be carefully controlled and the optimal content was determined to be 1% (w/w) [29].

Polymer FET sensors toward alcohols pay much attention on sensing mechanism. Many valuable conclusions have been achieved: (1) Sensing response is related with alcohol molecular volume which affects the analyte diffusion into the polymer film [39]. Alcohol with smaller molecular volume will be easier to diffuse into the film. (2) Polar-type interaction dominates in polar-polymer (for example, Poly-DPOT with alkoxy chain) sensor which makes the device more sensitive toward polar alcohols. On the other hand, dispersion-type interaction is recognized in non-polar-polymer (for example, Poly-DDT with alkyl chain) sensor which is more sensitive to long alkyl chain-bearing alcohols [31,32]. (3) Alcohols can trap the hole charge carriers in the channel region due to the lone electron pair in the hydroxyl group (–OH) [17,30,34]. The negative shift of threshold voltage is ascribed to the deep trap states induced by the hydroxyl group in the alcohols. The mobility decrease (Δ*μ*) is more easily interrupted by shallow trap density generated from weak interaction between gas analytes and polymers. Both deep trap and shallow trap states can be erased by desorption of gas analytes [29].

### 2.4. Other Gas Sensors

**Figure 5:**
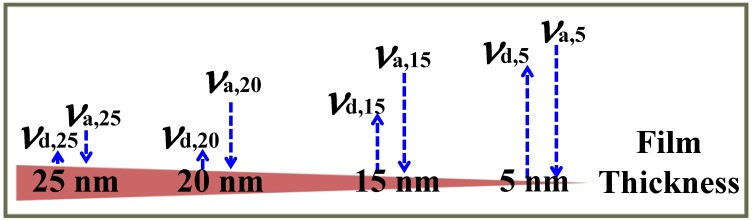
The supposed sensing process of H_2_S in film of polymer PSFDTBT. Both adsorption and desorption speeds of H_2_S molecules increase when the polymer films become thinner. However, desorption speed (*ν*_d,n_; n = 25, 20, 15, and 5 nm) increases much faster than adsorption speed (*ν*_a,n_). The length of arrows denotes the level of the speed.

H_2_S is a toxic and flammable gas with a characteristic odor of rotten egg. Concentration of H_2_S is usually lower than 1 ppb in unpolluted areas and 5 ppb H_2_S for long-time exposure can induce adverse health effects including respiratory, eye and nasal problems. Little work has been carried out on H_2_S gas sensors but one excellent OFET sensor based on spiro-annulated polymer PSFDTBT is reported by Lv and her coworkers [33]. In this work, the H_2_S sensor exhibited high sensitivity, excellent selectivity, fast response, and good operational stability. Under applied voltage of *V*_DS_ = *V*_GS_ = −30 V, the lowest concentration that could be detected was only 1 ppb, at which the current decrease (Δ*I*_SD_) was still 35% high. Polymer PSFDTBT device is to date the most sensitive H_2_S sensor based on organic semiconducting film. Investigation on the sensing process shows that the sensitivity is greatly related with the film thickness. The sensitivity increases first and then decreases as polymer films become thinner. Further experiment proves that, by thinning the active layer, both analyte adsorption and desorption speeds increase but desorption speed rises faster than the adsorption speed (Figure 5). The optimized film thickness is found to be 20 nm.

**Scheme 4:**
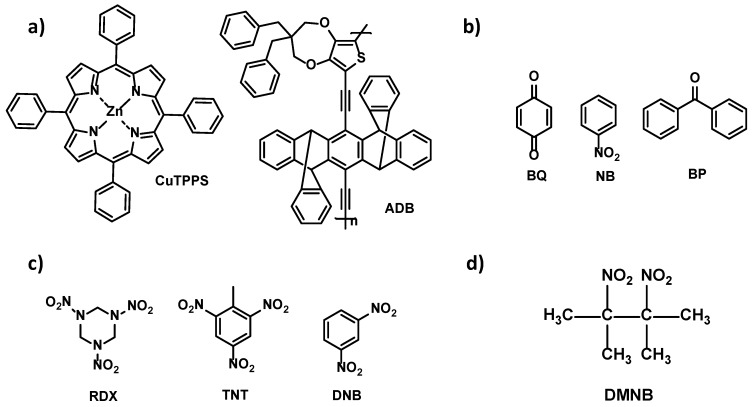
Chemical structures of: (**a**) compounds CuTPPS (CuII tetraphenylporphyrin) and ADB (copolymer of diethynyl-pentiptycene and dibenzyl-ProDOT); (**b**) non-explosive and (**c**) explosive nitro-based gas analytes; and (**d**) 2,3-dimethyl-2,3-dinitrobutane (DMNB).

Nitro based explosive gases, e.g., 1,3,5-trinitro-1,3,5-triazacyclohexane (RDX), 2,4,6-trinitrotoluene (TNT), and dinitrobenzene (DNB), were successfully detected by a ternary composite film of P3HT, CuTPP (CuII tetraphenylporphyrin) and ADB (copolymer of diethynyl-pentiptycene and dibenzyl-ProDOT) as shown in Scheme 4 [35]. This sensor showed ignorable response toward non-explosive nitro based gases like nitrobenzene (NB), benzoquinone (BQ) and benzophenone (BP), indicating an excellent selectivity for nitro based explosive analytes such as TNT, RDX, DNB. Even 10 ppb TNT gas gave an obvious current increase (–Δ*I*_SD_) of around 18% at *V*_DS_ = −40 V and *V*_GS_ = −20 V. In this ternary film, compound ADB makes the film porous and rough which eventually increases the surface-to-volume ratio; CuTPP can form a coordinate bonding between the metalloporphyrin molecule and the nitro group [48] as well as the π-stacking between the porphyrins and the aromatic rings from P3HT polymer. The strong interaction between electron deficient RDX, TNT and DNB gases and electron rich porphyrin macrocycle improves the film conductance, which should be responsible for the high sensitivity toward explosive nitro based gases. 2,3-dimethyl-2,3-dinitrobutane (DMNB), which is usually blended with commercial explosives, is used as a detection taggant for explosives. OFET sensors based on the semiconducting polymer PBTTT were able to detect 6.5 ppm DMNB. After exposure to DMNB gas, the source-drain current shows an obvious increase. Additionally, the largest current increase (–Δ*I*_SD_) is 36.5% at *V*_DS_ = −100 V and *V*_GS_ = −53 V [26]. Interaction between DMNB and PBTTT has not been clearly explained in this experimental work and more work should be carried to understand the sensing mechanism.

Dimethyl methylphosphonate (DMMP) OFET sensor is just mentioned in a report about chemiresistor sensor [36,49]. In this Experiment, the mixture of hexafluoroisopropanol substituted polythiophene (HFIP-PT) and single-walled carbon nanotube acted as the sensing layer. Under exposure of DMMP saturated vapor gas, the source-drain current (*I*_SD_) reduced and the threshold voltage shifted negatively. No more discussions on OFET sensor are presented in this work. CHCl_3_ gas was under detection of poly(3-butylthiophene) based OFET sensor, where CHCl_3_ proved to increase the flowing current in the channel [37]. There are no discussions on the sensing process and therefore much more work is needed to clarify the interaction between CHCl_3_ gas and semiconducting polymer.

Even structurally similar isomers can be differentiated through use of multi-parameter. Xylene, including three isomers which are *o*-xylene, *m*-xylene and *p*-xylene, impair the human respiratory system, the central nervous system, liver, kidneys, eyes, and skin. An OFET sensor based on an ambipolar semiconducting polymer PDPPHD-T3 could detect and discriminate the three xylene isomers at 40 ppm with applied voltages of *V*_SD_ = −70 V and *V*_GS_ = −60 V [38]. Upon exposure to the three xylene isomers, both the hole mobility (*μ*_h_) and electron mobility (*μ*_e_) decreased. Based on the measured transfer curve, multi sensing parameters which were changes in hole voltage threshold (Δ*V*_th-h_), normalized changes in the hole mobility (Δ*μ*_h_/*μ*_hb_), hole subthreshold swing (Δ*SS*_h_), changes in the electron voltage threshold, (Δ*V*_th-e_), normalized changes in the electron mobility (Δ*μ*_e_/*μ*_eb_) and electron subthreshold swing (Δ*SS*_e_) were calculated. Three xylene isomers could then be clearly distinguished from each other using a principle component analysis (PCA) plot of six sensing parameters in response to different xylene isomers at 40 ppm. DFT calculation shows that both HOMO and LUMO energies increase after adsorption of xylene isomers. This indicates a decrease in the hole-injection barrier and increase in the electron-injection barrier, which agrees well with the experimental sensing response of PDPPHD-T3 toward xylene isomers. Additionally, swelling of the polymer film induced by the adsorption of the xylene isomers affects the change of the sensing parameters. FTIR spectroscopy has experimentally monitored the shift of stretching vibrations including C=O, C–N, C=C, C–C and C–H bonds, proving the complexes of PDPPHD-T3 polymer and xylene isomers.

**Figure 6:**
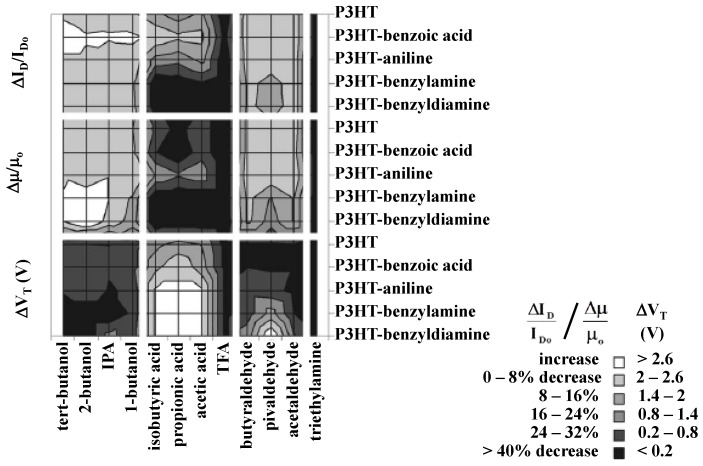
Response of five polymers toward twelve gas analytes in the sensor array. Baseline is established through operation in ambient conditions, and sensor response is measured after exposure to 40 ppm of each analyte for 2 min. Reprinted with permission from Ref. [39]. Copyright (2006) American Institute of Physics.

Due to the advantage of multi-parameter extracted from transfer characteristics, transistor sensors can diagnose more than one analyte at the same time. Multi-parameter data provide a rich source of sensing information and can reveal the non-obvious features of weak interaction between gas analytes and polymer semiconductors. A PTA-OMe sensor has recorded the change of four parameters *V*_T_, *μ*, *I*_ON_, *I*_OFF_ at *V*_GS_ = *V*_DS_ = ±2.5 V when the transistors are under exposure of different alcohols [34]. The PTA-OMe had a quite low hole mobility around 5 × 10^−5^ cm^2^·V^−1^·s^−1^ and a weak response toward alcohols. However, with the four measured parameters, sensing performance of four tested alcohols (methanol, ethanol, 1-propanol and isopropanol) could be discriminated. Sensor arrays with integrated OFET sensors show much better performance. For example, an array comprising four sensors with permutations of 70 nm and 140 nm film thicknesses and polybutylthiophene and polyoctylthiophene were fabricated [27]. This array could differentiate the mixtures of triethylamine and hexylamine by measuring the variation of *V*_T_, *I*_SD_, and *μ*. Another integrated sensor array, composed of five polymers with complementary sensing response by incorporating functional groups into the molecules, was fabricated by inkjet printing method [39]. Twelve gas analytes were exposed to this sensor array and the response was summarized in Figure 6, in which three parameters (Δ*I*_SD_, Δ*μ*, and Δ*V*_T_) were recorded. The differential response of multi-parameter makes these OFETs attractive for use in electronic nose.

Existence of water vapor in the ambient environment is inevitable. In order to realize the application of OFET sensor out of lab condition, it is important to study the water vapor effect toward OFET sensor. Most of the reported sensing polymer semiconductors have good air stability and show ignorable effect toward water vapor [13,14,22,33,50]. However, the water effect should be minimized so that a pure analyte effect can be achieved. For example, 60% RH in air and 85%–90% RH in water vapor were studied before exposure of ammonia to the P3HT OFET device [13]. The source-drain current showed a little increase. To get pure ammonia gas effect, this water effect was then eliminated by subtracting the net current difference in air (60% RH) and in water vapor (85%–90% RH). In addition, mixing P3HT with polystyrene also greatly improved the device stability by preventing the water from diffusing into the dielectric/semiconductor directly [22]. The most important factor to minimize the water effect is the air stability of OFET device, which can be enhanced by modification of self-assembled monolayer on dielectric layer and usage of air-stable sensing semiconductors [8].

## 3. PFET Sensors with Dielectric Layer as Sensing Layer

Analyte exposure upon an OFET changes the source-drain current flowing in the first few monolayers above the dielectric/semiconducting polymer interface. Therefore, direct functionalization on the dielectric layers efficiently controls the sensing response, which further proves the interaction between gas analytes and semiconducting polymer/dielectric interface. In an OFET sensor, the dielectric layer can influence the sensing response from two sides: (1) film morphology of semiconducting polymer related to analyte adsorption; and (2) the flowing current in the conducting channel by the analyte/dielectric interaction. Moreover, current increase or decrease induced by the same gas analyte is dependent on the dielectric/semiconducting polymer interface [18,51,52].

Morphology optimization of polymer semiconductors greatly enhances their sensing response toward gas analytes [25]. Polymer PBTTT was separately spin-coated onto three kinds of surfaces that were bare, octyltrichlorosilane (OTS) and CYTOP modified SiO_2_/Si surface. After NH_3_ exposure, PBTTT film on OTS, with the highest film crystallinity among the three dielectrics, exhibited apparently the largest current decrease. Optimal film morphology to achieve both high surface-to-volume ratio and high mobility charge carrier is important in good gas sensing characteristics.

**Scheme 5:**
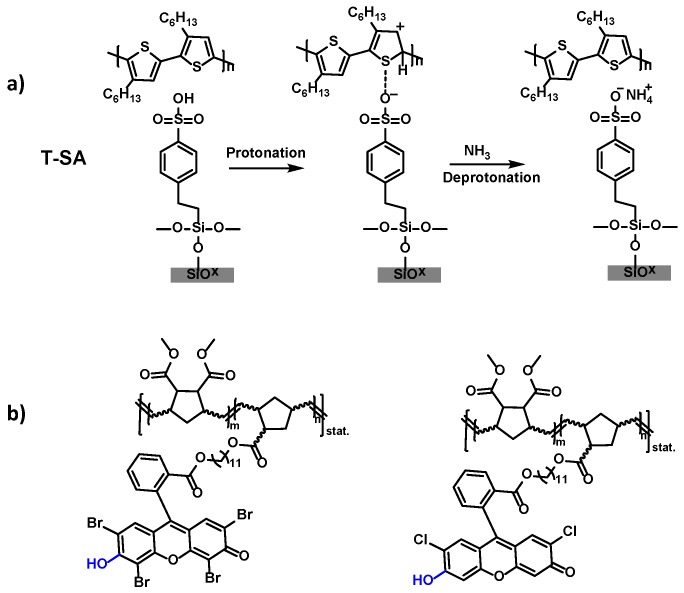
(**a**) Proposed sensing mechanism with siloxane (T-SA) modified dielectric as the sensing layer; and (**b**) chemical structures of ROMP-dielectric materials: (left) statistic co-polymer with eosin Y as NH_3_-sensitive group (m = 300, n = 3), and (right) statistic co-polymer with 2,7-dichlorfluorescein as NH_3_-sensitive group (m = 300, n = 3).

Siloxane layer (T-SC/SA) bound with sulfonic acid group was modified onto the SiO_2_/Si surface (Scheme 5a) [19]. In a BGTC structured device, the sulfonic acid group protonated the semiconducting P3HT and resulted in high positive *V*_T_ of up to 45 V. Under exposure to alkaline amines, the positive *V*_T_ showed negatively shift of about 60 V due to the deprotonation effect of the alkaline amines. In contrast, a negative shift of *V*_T_ was only 10 V for the bare SiO_2_/Si dielectric and was around 30 V for n-hexadecyltrichlorosilane (HDTS)/SiO_2_/Si dielectric. The huge electric response of T-SC/SA to alkaline amines holds high promise for its applications in chemical sensors. Ring-opening metathesis polymerized (ROMP, Scheme 5b) dielectric materials exhibited similar effect as T-SC/SA dielectric and TGBC structure was used in this study [15]. When exposed to ammonia, the –OH group of ROMP dielectric could be deprotonated and thus formed mobile ammonium ions (NH_4_^+^). Under applied voltage, the mobile NH_4_^+^ ions could move within the dielectric materials toward drain electrode and localized near the dielectric/P3HT interface or even in the P3HT layer. Hence, the density of mobile charge carriers in the channel was increased by the protonated NH_3_ by the ROMP dielectric layer.

Metal gate could be replaced with conducting polymer sensing layer, usually in a MOSFET sensor. Much work on conducting polymer gate FETs for vapor sensing has been reported. There are already a few reviews to summarize this topic [53,54,55]; therefore, we will not further discuss them here.

## 4. Conclusions and Future Perspectives

This review summarizes the PFET sensors categorized by sensing layers consisting of semiconducting layer, dielectric layer and conducting gate layer, separately, with the focus on sensing polymers, gas analytes, sensor performance and sensing mechanism. OFETs with polymers as the semiconducting layer have been demonstrated as effective sensors in gas detection. Although discussions on analyte–polymer interaction have been addressed, there is still a lack of systematic rules to predict and instruct the fabrication of OFET sensors. More gas analytes and sensing polymers need to be introduced, which will enrich the sensing database and help to understand the sensing mechanism. OFET sensor array is able to analyze complex analytes and is a promising area in the future. Furthermore, humidity in the air can impair the OFET sensor and this effect should be minimized. Although calibration of sensor measurements by subtracting the humidity effect at various humidity levels is available, improving the stability of sensing polymers is the most efficient approach, and needs much more investigation. Other approaches including mixing sensing polymer semiconductors with dielectrics and modifying the dielectric layers with self-assembled layers should also be developed.

## Figures and Tables

**Table 1:** Summary of the sensing polymer semiconductors and the key parameters of PFET sensors.

Materials	Analytes	/ppm	Device	–*V*_DS_/V	–*V*_GS_/V	Response Time	Reference
P3HT	NH_3_	100%	BGBC	4	4	10 min	[11]
	NH_3_	0.03–1	Vertical	1	1	-	[12]
	NH_3_	0.1–25	BGBC	60	30	5–25 s	[13]
	NH_3_	16–198	BGBC	1	1	52 s (99 ppm)	[14]
	NH_3_	100	BGBC	100/10	85	600 s	[15]
	NO_2_	0.038–25	BGBC	50	50	5 min	[16]
	ethanol acetone	500–7000	BGBC	60	60	8–15 min	[17]
	ethanol methanol	SVP ^3^	TGTC	2	0.8	-	[18]
	NH_3_ diethylamine triethylamine	-	BGTC	45	60	0.5–2 h	[19]
P-29-DPP-SVS	NH_3_	29–1000	BGTC	5	5	5 min	[20]
pDPPCOOH-BT	NH_3_	0.01–1000	BGBC	60	80	50 s	[21]
	1,4-diaminobutane, Et3N, piperidine	10 ppm	BGBC	60	80	50 s	[21]
P3HT/PS	NH_3_	5–50	BGTC	40	40	5–10 min	[22]
PQT-12	NH_3_	0.1–15	BGBC	90	corona charge	≥5 min	[23]
polyaniline	NH_3_	1–20	BGBC	8	10	10 s	[24]
PBTTT	NH_3_	10–100	BGTC	30	30	40 s	[25]
	DMNB	6.5	BGTC	100	53	10 min–20 h	[26]
Array sensor of Poly-3-butylthiophene Poly-3-octylthiophene	octylamine butylamine	8.9	BGBC	20	20	900 s	[27]
	hexylamine triethylamine	30	BGBC	20	20	900	[27]
PTA-F	NO_2_	0.01–50	BGTC	3	3	20 s	[28]
PTA-OMe							
DPP-SVS/GO ^1^	ethanol	SVP ^3^	BGTC	30	30	20 s	[29]
F8T2/GO	ethanol acetone	SVP ^3^	BGTC	80	80	10 min	[30]
Poly-DPOT	Ethanol 1-hexanol	700	BGBC	100	40	2 min	[31,32]
Poly-DDT							
PSFDTBT	H_2_S	0.001–1	BGTC	30	30	5–15 s	[33]
PTA-OMe	MeOH	10^3^–10^6^	BGTC	3	3	2–3 min	[34]
	EtOH						
	PrOH						
	IPA						
P3HT/CuTPP/ADB	RDX	<0.01	BGBC	40	20	20 min	[35]
	TNT						
	DNB						
HFIP-PT/SWCNT ^2^	DMMP	SVP ^3^	BGBC	0.001	5	10 min	[36]
poly-butylthiophene	CHCl_3_	SVP ^3^	BGTC	10	3	-	[37]
PDPPHD-T3	*o*-xylene	40-320	BGBC	70	60	12 s	[38]
	*m*-xylene						
	*p*-xylene						
Sensor array of P3HT P3HT-benzoic acid P3HT-aniline P3HT-benzylamine P3HT-benzyldiamine	tert-butanol	40	BGBC	20	20	2 min	[39]
	2-butanol						
	isopropanol						
	1-butanol						
	isobutyric acid						
	propionic acid						
	acetic acid						
	TFA						
	butyraldehyde						
	pivaldehyde						
	acetaldehyde						
	triethylamine						

^1^ GO is the abbreviation of graphene oxide; ^2^ SWCNT is the abbreviation of single-walled carbon nanotube; ^3^ SVP indicates the saturated vapor pressure at room temperature.
